# Role of mitochondrial quality control in neurodegenerative disease progression

**DOI:** 10.3389/fncel.2025.1588645

**Published:** 2025-05-20

**Authors:** Tingting Liu, Weibo Sun, Shuhao Guo, Zhiying Yuan, Minghang Zhu, Jing Lu, Tao Chen, Yuanyuan Qu, Chuwen Feng, Tiansong Yang

**Affiliations:** ^1^Heilongjiang University of Chinese Medicine, Harbin, China; ^2^Department of Breast Surgery, Harbin Medical University Cancer Hospital, Harbin, China; ^3^Rehabilitation Department II, The First Affiliated Hospital of Heilongjiang University of Chinese Medicine, Harbin, China; ^4^Key Laboratory of Chinese Medicine Informotics in Heilongjiang Province, Harbin, China

**Keywords:** mitochondrial quality control, Alzheimer’s disease, Parkinson’s disease, Huntington’s disease, amyotrophic lateral sclerosis

## Abstract

Neurodegenerative diseases are a diverse group of neurological disorders, in which abnormal mitochondrial function is closely associated with their development and progression. This has generated significant research interest in the field. The proper functioning of mitochondria relies on the dynamic regulation of the mitochondrial quality control system. Key processes such as mitochondrial biogenesis, mitophagy, and mitochondrial dynamics (division/fusion) are essential for maintaining this balance. These processes collectively govern mitochondrial function and homeostasis. Therefore, the mitochondrial quality control system plays a critical role in the onset and progression of neurodegenerative diseases. This article provides a concise overview of the molecular mechanisms involved in mitochondrial biogenesis, mitophagy, and mitochondrial dynamics, explores their interactions, and summarizes current research progress in understanding the mitochondrial quality control system in the context of neurodegenerative diseases.

## 1 Introduction

Mitochondria, as quintessential organelles in eukaryotic cells, serve dual roles as cellular energy suppliers and key regulators of calcium homeostasis, oxidative stress responses, and apoptotic signaling pathways (Kamer and Mootha, 2015; Mattson et al., 2008; Zhou and Tian, 2018). Neurons, characterized by exceptionally high metabolic demands, critically depend on mitochondrial bioenergetic output for both normal functioning and survival (Zhou and Tian, 2018). Consequently, mitochondria are indispensable for the sustenance of neuronal life, and their robust activity and functional integrity are crucial for the preservation of neuronal structure and vitality.

Neurodegenerative diseases (NDDs), also known as neurodegenerative diseases, are a class of disorders characterized by the progressive degeneration of neuronal structure or function in the central or peripheral nervous systems (Wilson et al., 2023). Because these diseases are incurable malignant diseases, they adversely affect the lives of millions of people worldwide. Common clinical neurodegenerative diseases include Alzheimer’s disease (AD), Parkinson’s disease (PD), Huntington’s disease (HD), amyotrophic lateral sclerosis (ALS), etc. More and more researchers are committed to the related research of neurodegenerative diseases. Although NDDs have been studied for many years, its pathogenesis is still not fully elucidated due to the complexity of pathogenic factors. At present, several studies have shown that patients with neurodegenerative diseases have some common pathological characteristics in the early stage of the disease: pathological protein aggregation and mitochondrial damage or mitochondrial dysfunction in vulnerable brain regions (Klemmensen et al., 2024; Lin and Beal, 2006; Wang D. X. et al., 2021), and the normal operation of mitochondrial function depends on the dynamic regulation of the mitochondrial quality control (MQC) system (Zheng et al., 2019). Emerging research demonstrates that the mitochondrial quality control (MQC) system maintains cellular homeostasis through coordinated processes including mitochondrial biogenesis, mitophagy, and mitochondrial dynamics (fission/fusion). Dysregulation in any of these regulatory nodes may induce ultrastructural mitochondrial abnormalities and functional impairments, ultimately precipitating axonal degeneration and neuronal apoptosis—pathological hallmarks that define neurodegenerative pathogenesis (Hong et al., 2024). Therefore, maintaining the balance of the mitochondrial quality control system is essential for neuronal activity in the brain of neurodegenerative diseases.

In conclusion, in this review, we briefly describe the mitochondrial structure, summarize the specific regulatory mechanisms of the mitochondrial quality control system and the interaction between various pathways, and finally elaborate the research progress of mitochondrial biogenesis, mitophagy and mitochondrial dynamics in several common neurodegenerative diseases, in order to understand more about the mitochondrial quality control system and provide new research targets and ideas for the treatment of neurodegenerative diseases.

## 2 Mitochondrial structure

**FIGURE 1:**
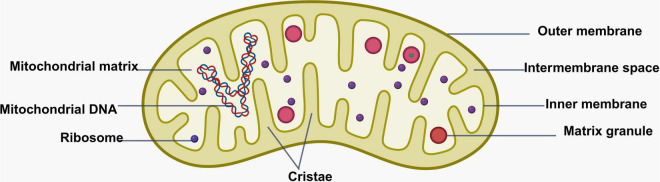
Schematic diagram of the mitochondrial structure. Figure was created with BioRender software.

Mitochondria are double-membrane-bound organelles. The membrane is primarily composed of lipids and proteins. Due to the special characteristics of the membrane components, it promotes the bending and flow of the membrane. The two-layer membrane divides mitochondria into multiple cavities, which is known as “mitochondrial compartmentalized structure.” The mitochondrial structure mainly includes four distinct regions, including mitochondrial outer membrane (Outer Mitochondrial Membrane—OMM), mitochondrial inner membrane (Inner Mitochondrial Membrane—IMM), mitochondrial matrix, and intermembrane space (Kramer and Bressan, 2018). The OMM is permeable, mediates mitochondrial signal transmission, and carries molecules involved in fusion and fission, playing a crucial role in mitochondrial dynamics (Lin et al., 2022). IMM is composed of inner boundary membrane and mitochondrial cristae, and the inner boundary membrane is parallel to the outer mitochondrial membrane. Mitochondrial cristae are formed by multiple folds of the inner mitochondrial membrane extending into the mitochondrial matrix (Pape et al., 2020), They include crista junctions, which connect the cristae membranes to the inner boundary membrane, and cristae tips at the distal ends of the cristae membranes (Kondadi et al., 2020). The mitochondrial matrix contains enzymes, proteins (including mtDNA, RNA), ribosomes, and metabolites critical for the TCA cycle and fatty acid oxidation (Kaasik et al., 2007; Yan et al., 2019). Alterations in the structure and function of mitochondrial compartments affect mitochondrial homeostasis and quality control (Iovine et al., 2021), which are closely linked to neurological diseases (Figure 1).

## 3 Mitochondrial quality control

Mitochondrial quality control refers to the dynamic and coordinated cycle of regulatory processes that maintain mitochondrial function. It is an endogenous cellular protective program essential for maintaining mitochondrial homeostasis and function in eukaryotes (Gan et al., 2018; Sedlackova and Korolchuk, 2019). MQC involves a variety of mechanisms to regulate and initiate the corresponding repair mechanism according to the degree of mitochondrial damage, which mainly includes mitochondrial biogenesis, mitochondrial dynamics (division/fusion), and mitophagy. The three interact with each other to maintain mitochondrial homeostasis or balance of mitochondrial quality control system.

### 3.1 Molecular mechanisms of mitochondrial biogenesis

Mitochondrial biogenesis is a strictly regulated process. Under the joint regulation of existing mitochondrial nuclear DNA (nDNA) and mitochondrial DNA (mtDNA), new mitochondria are synthesized to replace damaged mitochondria, so as to repair mitochondrial structure, maintain mitochondrial function, increase antioxidant effect, reduce pathological oxidative stress and promote ATP production to meet the metabolic needs of eukaryotic cells under physiological and pathological conditions (Ferreira et al., 2019; Popov, 2020; Zhou et al., 2021).

**FIGURE 2:**
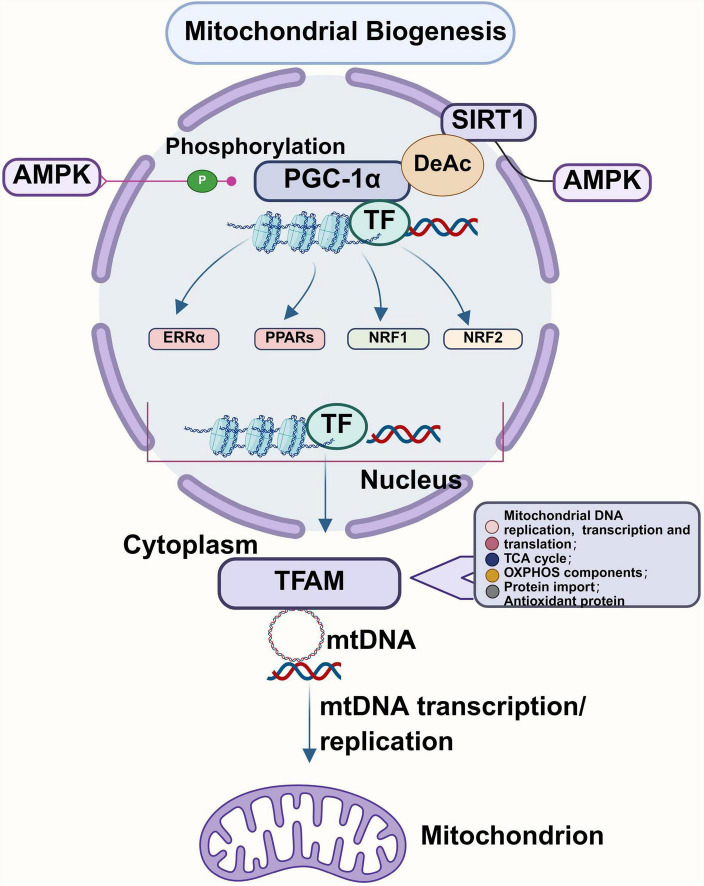
Schematic illustration of the mitochondrial biogenesis. PGC-1α serves as the primary modulator of mitochondrial biogenesis, with SIRT1 and AMPK facilitating its activation through deacetylation and phosphorylation, respectively. This activation enhances the expression and functionality of TFAM by stimulating the activity of PPARs, NRF1, NRF2, and ERR-α. Subsequently, TFAM binds to the promoter region of mitochondrial subunits, thereby promoting the replication and transcription of mitochondrial mtDNA. Figure was created with BioRender software.

Mitochondrial biogenesis (Mitochondrial biogenesis, MB), as a way of mitochondrial self-renewal, regulates the number, size, and quality of mitochondria (Ploumi et al., 2017). Different stress stimuli regulate mitochondrial biogenesis. The synthesis of mitochondrial membrane, mtDNA replication, transcription, translation, and the synthesis and import of mitochondrial proteins encoded by nuclear DNA are important factors in the process of mitochondrial biogenesis (Golpich et al., 2017; Zhang and Xu, 2016). Markers of mitochondrial biogenesis are mtDNA copy number, elevated mtDNA/nDNA ratio, and mitochondrial gene expression level (Andres et al., 2017). This section mainly introduces the key regulator peroxisome proliferator-activated receptor-gamma coactivator-1alpha (Peroxisome-proliferator-activated receptorγcoactivator-1α, PGC-1α), nuclear respiratory factor (Nuclear respiratory factors 1/2, NRF1/2), mitochondrial transcription factor A (Mitochondrial transcription factor A, TFAM) mediates the regulatory mechanism of mitochondrial biogenesis (Figure 2).

As a key transcriptional coactivator, PGC-1α primarily regulates mitochondrial biogenesis. It is highly expressed in energy-demanding tissues (e.g., heart, brain, skeletal muscle) (Cheng et al., 2018). PGC-1α is often referred to as “the main regulator of metabolism” or “the initiator of metabolic molecules” (Villena, 2015). It acts as a coactivator for a variety of nuclear receptors and transcription factors, participating in the regulation of glucose metabolism, oxidative phosphorylation, overall energy homeostasis, and the genetic program of antioxidant production (Austin and St-Pierre, 2012; Wang et al., 2019b). Research has indicated that the AMPK-PGC-1α-Nrf-TFAM axis and the AMPK-SIRT1-PGC-1α-Nrf-TFAM axis are two major pathways that regulate mitochondrial biogenesis. The AMPK/SIRT1/PGC-1α signaling pathway is crucial for controlling mitochondrial biogenesis and plays a significant role in stabilizing cellular energy metabolism (Yan et al., 2020). During mitochondrial biogenesis, the upstream signal regulators, silent information regulator 1 (SIRT1) and adenosine monophosphate-activated protein kinase (AMPK), activate PGC-1α through phosphorylation and deacetylation modifications. This activation then stimulates PPARs, nuclear respiratory factors 1 and 2 (Nrf1/2), and estrogen receptor-related receptors α (ERRα), enhancing the expression and activity of TFAM (Fontecha-Barriuso et al., 2020; Peng et al., 2022). TFAM subsequently binds to the promoter region of the mitochondrial subunit, promoting mitochondrial mtDNA replication and transcription.

The nuclear respiratory factor (Nrf1/2), functioning as a conserved transcriptional activator, predominantly localizes within skeletal muscle, brain tissue, and lung tissue (Gleyzer et al., 2005; Kiyama et al., 2018; Klinge, 2017). Positioned downstream of PGC-1α, a pivotal regulator coordinating mtDNA and nuclear DNA, Nrf1/2 serves as the primary initiator of mitochondrial biogenesis. It orchestrates mtDNA transcription and replication by stimulating the transcription of TFAM (Wong-Riley, 2012). These nuclear respiratory factors are primarily tasked with governing the expression of mitochondrial respiratory genes (proteins), key mitochondrial enzyme transcription, coding genes for mitochondrial respiratory complex subunits, mitochondrial translation components, and heme biosynthase (Dinkova-Kostova and Abramov, 2015; Esteras and Abramov, 2022). Their absence results in defects in mitochondrial biogenesis and mitochondrial protein import, ultimately leading to cell death (Kiyama et al., 2022; Klinge, 2017). Nrf1/2 maintains mitochondrial homeostasis by modulating the nuclear gene expression of components within the oxidative phosphorylation system (Scarpulla, 2002a,b). Mitochondrial transcription factor A (TFAM) is a nuclear gene-encoded mitochondrial transcription factor that binds upstream of the transcription start site and plays an important role in the replication, transcription, assembly, and maintenance of the stability of mtDNA (Kang et al., 2018; Kukat et al., 2015). TFAM binds to mtDNA in both specific and non-specific ways, with specific binding to TFAM being essential for initiating mitochondrial transcription. TFAM proteins bind to the light and heavy chain promoter regions of DNA (Zorzano and Claret, 2015), increasing the flexibility of DNA and promoting its compression (Farge et al., 2012). This binding also recruits mitochondrial RNA polymerase to initiate mtDNA transcription (Malarkey et al., 2016). However, the mechanism of TFAM’s compression of mtDNA is currently unclear. TFAM binds non-specifically to all sequences of mtDNA, wrapping around the mtDNA structure to form stable protein patches. Under pathological conditions, disruption of TFAM can lead to mtDNA mispackaging, a massive release of damaged mtDNA, and down-regulation of TFAM also reduces the stability of the mtDNA mimetic nuclear structure (West et al., 2015). TFAM is almost exclusively present in a DNA-bound form (Takamatsu et al., 2002), and degradation of TFAM proteins by mitochondrial matrix proteases directly affects mtDNA copy number (Matsushima et al., 2010). In summary, TFAM protein abundance is closely related to mtDNA content, and TFAM affects mitochondrial biosynthesis by regulating mtDNA copy number through its interaction with mtDNA (Picca and Lezza, 2015).

In conclusion, mitochondrial biogenesis plays an important role in maintaining the health of the mitochondrial network and rescuing mitochondrial dysfunction. Activating mitochondrial biogenesis is a potential avenue for the treatment of mitochondrial-related diseases.

### 3.2 Molecular mechanisms of mitophagy

Mitophagy, which may be described as a process of “eating itself,” is the selective isolation of excess or damaged mitochondria by autophagosomes, which are then degraded by lysosomes (Pickles et al., 2018). Mitophagy is classified into three main categories according to the physiological conditions under which it occurs: basal mitochondrial autophagy, stress-induced mitochondrial autophagy, and programmed mitophagy. The process of mitochondrial autophagy can be broadly divided into three stages: preparation, initiation and degradation (Yamashita and Kanki, 2017). In the early stages, mitochondrial damage leads to the depolarization of the membrane potential. This triggers the activation of autophagy-associated proteins, which form a bilayer membrane upon receiving initiation signals. The bilayer membrane then wraps around the damaged mitochondria. Once the bilayer membrane has been stretched, it begins to close the loop at a slow rate, forming mitochondrial autophagosomes. Autophagosomes fuse with lysosomes to form mature autophagolysosomes. Lysosomal hydrolases degrade the autophagosomal contents, completing the process of mitophagy (Chen and Chan, 2009).

The scholarly exploration into the regulatory mechanisms of mitophagy has garnered significant interest. Presently, the identified pathways that facilitate mitophagy are predominantly categorized into ubiquitin (Ub)-dependent and non-ubiquitin (Ub)-dependent systems (Palikaras et al., 2018). The Ub-dependent processes encompass PINK1/Parkin-mediated mitophagy and ubiquitin-regulated mitophagy, while the non-Ub-dependent processes involve receptor protein and lipid-mediated mitophagy (Fritsch et al., 2020). The receptor proteins predominantly engaged in mitophagy include BNIP3L/NIX, FUNDC1, FKBP8, PHB2, and AMBRA1 (Yao et al., 2021). This section will examine the regulatory mechanisms of PINK1/Parkin-dependent mitophagy, as well as those of BNIP3L/NIX and FUNDC1-mediated mitophagy.

#### 3.2.1 PINK1/Parkin mediated mitophagy

The pathway consisting of PINK1 and Parkin is a key participant in mitophagy, as well as the most common and classical pathway of autophagic mitochondria (Cai and Jeong, 2020; Lazarou et al., 2015). PINK1 is a serine/threonine kinase located in mitochondria as a highly conserved protein. Mitochondrial protein associated with both outer and inner membranes (Khaminets et al., 2016). Parkin is an E3 ubiquitin ligase, diffusely distributed in cytoplasmic lysate in a state of self-repression (Wang et al., 2023). Parkin plays an important role in mitochondrial autophagy signaling as a downstream factor of the PINK1-mediated mitophagy pathway. PINK1 and Parkin interact to mediate mitophagy, thereby maintaining mitochondrial homeostasis. The loss or reduced function of both proteins leads to mitochondrial damage. The manner in which damaged mitochondria activate mitophagy is determined by three major factors: PINK1, Parkin and ubiquitin chain.

As a molecular sensor of mitochondrial damage, PINK1 accumulates on the outer mitochondrial membrane due to impaired transmembrane translocation upon mitochondrial membrane potential (ΔΨm) depolarization (Lu et al., 2023). Mediated by the TOM complex, PINK1 undergoes trans-autophosphorylation and subsequent conformational rearrangement, thereby activating the E3 ubiquitin ligase activity of Parkin through its transition from an autoinhibited state to an active conformation (Riley et al., 2013). Activated Parkin ubiquitinates outer mitochondrial membrane proteins including Mfn1, Mfn2, Miro1, and VDAC1 (Birsa et al., 2014; Geisler et al., 2010; Tanaka et al., 2010), followed by PINK1-mediated phosphorylation of these ubiquitinated substrates to amplify polyubiquitin chain signaling (Harper et al., 2018). Autophagy receptors (P62, NBR1, NDP52, OPTN, TAX1BP1) specifically recognize ubiquitin chains via their LC3-interacting regions, binding to microtubule-associated protein 1A/1B-light chain 3 (LC3) on autophagosomal membranes and directing the engulfment of damaged mitochondria. Ultimately, autophagosome-lysosome fusion generates autolysosomes, enabling programmed mitochondrial degradation (Turco et al., 2021).

#### 3.2.2 BNIP3/NIX mediated mitophagy

NIX, a protein with 50% homology to BNIP3 (Matsushima et al., 1998), binds to the mitochondrial outer membrane through its carboxyl transmembrane domain, thereby initiating mitophagy under ischemic or hypoxic conditions. BNIP3 and NIX engage in this process through two distinct mechanisms. Firstly, the N-terminus of BNIP3 interacts with LC3 or GABARAP, while NIX promotes hypoxia-induced mitophagy via phosphorylation at Ser81 (Yuan et al., 2017; Zhang et al., 2019). Secondly, the BH3 domain of BNIP3 may trigger the release of Beclin-1, thereby initiating mitophagy. Additionally, NIX has been identified as a substrate for Parkin, facilitating Parkin/PINK1-dependent mitophagy (Gao et al., 2015). Currently, the BNIP3/NIX complex is recognized as a critical factor in the removal of damaged mitochondria and the stimulation of mitophagy (Sandoval et al., 2008; Zhang and Ney, 2010). However, the precise mechanisms underlying their action remain to be elucidated.

#### 3.2.3 FUNDC1 mediated mitophagy

The FUN14 domain-containing protein 1 (FUNDC1) is widely present on the mitochondrial outer membrane and acts as a regulator of mitochondrial autophagy in response to hypoxia, inducing Parkin-independent mitophagy (Liu et al., 2012). In physiological conditions, where FUNDC1 is phosphorylated by tyrosine kinases, the induction of mitochondrial autophagy is prevented. Conversely, following hypoxia or loss of mitochondrial membrane potential, the inactivation or reduction of tyrosine kinase activity through the activation of PGAM5 will contribute to the downregulation of the phosphorylated FUNDC1 level. Consequently, FUNDC1 phosphorylation at Tyr18 and Ser13 undergoes dephosphorylation, resulting in a conformational change and elevation of the FUNDC1 affinity for LC3 or GABARAP. This, in turn, triggers the onset of mitochondrial autophagy (Zhang W. et al., 2017). In parallel, it has been documented that the phosphorylation of the FUNDC1-Ser17 site, mediated by ULK1, also initiates mitophagy (Liu et al., 2022; Zhou et al., 2017). These findings collectively indicate that FUNDC1 is a pivotal and indispensable receptor in the regulation of mitophagy under hypoxic conditions, and its function is modulated by phosphorylation during the mitophagy process.

**FIGURE 3:**
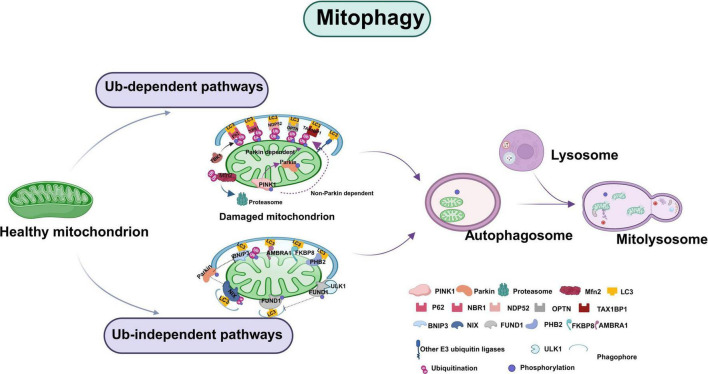
Schematic illustration of the mitophagy. Mitochondrial phagocytosis is facilitated by a complex interplay of mechanisms, primarily categorized into ubiquitin (Ub)-dependent and Ub-independent pathways. The Ub-dependent pathway is predominantly mediated by the PINK1/Parkin axis. Furthermore, a distinct set of mitophagy receptors is capable of directly interacting with LC3, obviating the need for extensive ubiquitination and thereby contributing to the Ub-independent pathway. The receptor proteins involved in mitochondrial autophagy include BNIP3L/NIX, FUNDC1, FKBP8PHB2, and AMBRA1, among others. Figure was created with BioRender software.

To encapsulate, mitophagy is an evolutionarily conserved mechanism that identifies and degrades dysfunctional mitochondria in a timely manner, thereby mitigating oxidative stress and enhancing cellular energy. Mitophagy adapts to various stress conditions and metabolic states, presenting distinct profiles. This process is a multifaceted and intricate regulatory mechanism, with multiple pathways mediating mitophagy demonstrating parallelism or redundancy to preserve the equilibrium between mitochondrial homeostasis and the mitochondrial quality control system (Figure 3).

### 3.3 Molecular mechanisms of mitochondrial dynamics

Mitochondria, as highly dynamic organelles, adapt to the extracellular and intracellular microenvironments by undergoing fusion and division, thereby regulating their position, size, and shape. These organelles not only provide energy for cellular functions but also ensure the proper operation of mitochondrial processes. Moreover, mitochondria play a crucial role in removing damaged mitochondria and in regulating mitochondrial respiration, calcium signaling, cell survival, apoptosis, and other vital cellular processes. This intricate physiological process is termed “mitochondrial dynamics” (Liesa et al., 2009; Tilokani et al., 2018; Zhou et al., 2021). Mitochondrial fusion is defined as the process of fusing the inner and outer membranes of two different mitochondria to form a new mitochondrion. This increases the resistance of the mitochondrial network and facilitates complementary interactions between damaged mitochondria (Gan et al., 2018; Szabo et al., 2018). Mitochondrial division serves to isolate damaged mitochondria and retain functionally intact mitochondria. The separated damaged mitochondria are further cleared by mitochondrial autophagy (Ni et al., 2015; Taguchi et al., 2007; Zheng et al., 2023). The balance between mitochondrial division and fusion is an important basis for normal cellular activity and the maintenance of energy homeostasis, as well as for neurons to perform normal functions (Flippo and Strack, 2017; Mishra and Chan, 2016). In the physiological state, mitochondrial fission and fusion are in dynamic equilibrium, with each process holding the other in check. However, disruption of this equilibrium, for example through the blockage of mitochondrial fusion and fission, may lead to oxidative stress, mitochondrial dysfunction and metabolic alterations, and ultimately to the development of mitochondria-associated diseases (Archer, 2013; Xiao et al., 2021). The regulatory mechanisms of mitochondrial dynamics are complex and involve multiple proteins and signaling pathways. In this part, we mainly summarize the regulatory mechanisms of mitochondrial dynamics key proteins such as Mitofusin 1 (Mfn1), Mitofusin 2 (Mfn2), Optic atrophy 1 (OPA1), Dynamin-related protein 1 (Drp1), and Fission protein 1 (Fis1) in mitochondrial fusion and fission.

#### 3.3.1 Mitochondrial fusion and regulatory mechanisms

Mitochondrial fusion is a cellular self-repair mechanism that combines healthy mitochondria with damaged or mutated mitochondria to repair the damage or compensate for the mitochondrial dysfunction caused by mutations through complementary effects, thus ensuring normal mitochondrial function and maintaining normal cellular physiological activities (Twig and Shirihai, 2011). The process of mitochondrial fusion involves the exchange of mtDNA, intermediates of the tricarboxylic acid cycle (TCA cycle), and respiration-related proteins. As a result, the new mitochondria formed after fusion have mtDNA pools, differences in membrane potential, and a diversity of proteins compared to the mitochondria before fusion (Zhou et al., 2021). Mitochondria are a two-layer membrane organelle. The process of mitochondrial fusion involves the fusion of the outer and inner mitochondrial membranes. The entire process of mitochondrial fusion can be briefly summarized as mitochondrial trans-bolus, mitochondrial outer membrane fusion, and mitochondrial inner membrane fusion (He and Maheshwari, 2023). Mitochondrial fusion is regulated by several membrane-anchored proteins, including Mfn1, Mfn2, and OPA1. Mfn1 and Mfn2 promote the fusion of the outer mitochondrial membrane (OMM), while OPA1 is the major mitochondrial DNA (mtDNA) in mammals responsible for the fusion of the inner mitochondrial membrane (IMM) and remodeling of the mitochondrial cristae (Cisneros et al., 2022; Lee H. et al., 2017; Sai et al., 2013; Wang et al., 2019a).

As key mitochondrial GTPases, Mfn1 and Mfn2 mediate the fusion of outer mitochondrial membranes (Tokuyama and Yanagi, 2023). They consist of an amino-terminal GTPase structural domain, two coiled-coil structural domains, and a double transmembrane structural domain embedded in the mitochondrial outer membrane (Chandhok et al., 2018). In mammals, Mfn1 and Mfn2 have analogous functions to a certain extent, with Mfn1 being expressed predominantly in the liver, adrenal glands, and heart, while Mfn2 is expressed primarily in the brain, heart, bone, and brown adipose tissue. Mfn1/2 is primarily localized to the outer mitochondrial membrane and endoplasmic reticulum surface, where it serves to anchor mitochondria to the endoplasmic reticulum and facilitate mitochondrial calcium uptake (Joaquim and Escobar-Henriques, 2020). The control of mitochondrial elongation is impacted by hypoxia, for instance. This process is regulated by Mfn1 deacetylation. In contrast, Mfn2 participates in mitochondrial fusion regulation. This is achieved through the formation of mitochondrial and endoplasmic reticulum contact sites (Basso et al., 2018; Oanh et al., 2017). Mfn2 exists in two distinct functional forms, the compressed inactive form and the expanded active form, which are associated with different biological roles (Franco et al., 2016). The activity of Mfn2 is also regulated by signal transducer molecule 2 (recombinant mothers against decapentaplegic homolog 2, Smad2), which acts as a scaffold to recruit Rab-Ras interacting factor 1 (RIN1) to form the Smad2-RIN1-Mfn2 complex with Mfn2, which ultimately promotes ATP synthesis and mitochondrial fusion (Kumar et al., 2016). Beyond mediating outer mitochondrial membrane fusion, Mfn1/2 participates in mitophagy regulation, mitochondrial cristae remodeling, and facilitates stress-associated unfolded protein response (UPR) (Hu et al., 2019; Tokuyama and Yanagi, 2023). In conclusion, Mfn1 and Mfn2 perform distinct functions during mitochondrial fusion in conjunction with one another, thereby influencing the pathogenesis of various diseases (Rosca et al., 2013).

OPA1 is a crucial protein involved in cristae remodeling. It is hydrolyzed by proteases in mitochondria to produce two forms: membrane-anchored L-Opa1 and processed S-Opa1 (Oma1, Yel1L, Parl) (Fry et al., 2023). Both isoforms are found in nearly equal amounts in physiological conditions (Annesley and Fisher, 2019; Del et al., 2018). Studies have indicated that the oligomerization of the long and short forms of OPA1 is responsible for the fusion or fission of the inner mitochondrial membrane (Dirks-Naylor et al., 2014). This process is initiated by the cleavage of L-Opa1 in the transmembrane structural domain, which results in the generation of S-Opa1. The soluble and cleaved form of OPA1, known as S-Opa1, is present in the intermembrane space (IMS). An excess of this form can result in mitochondrial fission and dysfunction, as demonstrated in research (Duan et al., 2023). In contrast, the L-Opa1 variant is anchored in the inner mitochondrial membrane. This form of OPA1 is involved in mitochondrial fusion, as evidenced by research (Civiletto et al., 2015; MacVicar and Langer, 2016). OPA1 is involved in mitochondrial cristae remodeling in addition to regulating IMM fusion. Studies have reported that overexpression of OPA1 reduces cristae width and causes tightening of mitochondrial cristae (Glytsou et al., 2016; Quintana-Cabrera et al., 2018). In the absence of OPA1, the inner mitochondrial cristae structure and inner membrane are damaged, which leads to the loss of mitochondrial membrane potential and ultimately affects mitochondrial function (Patten et al., 2014; Pernas and Scorrano, 2016).

#### 3.3.2 Mitochondrial fission and regulatory mechanisms

Mitochondrial fission refers to the process by which a mitochondrion divides into two mitochondria. This allows damaged or low-potential mitochondria to be separated from the network by mitosis, ensuring the maintenance of a healthy mitochondrial network (Zheng et al., 2020; Zhou et al., 2021). Mitochondrial division does not appear to be dependent on mitochondrial membrane potential. Abnormal mitochondrial fission results in mitochondrial fragmentation, triggering depolarization, oxidative stress, and ultimately severe neuronal damage (Barsoum et al., 2006; Chan, 2006; Chiang et al., 2015; Frank, 2006; Santos et al., 2015; Youle and van der Bliek, 2012). As previously noted, the regulatory genes Drp1 and Fis1 are essential for mitochondrial division. This process results in a decline in mitochondrial membrane potential, ultimately leading to mitochondrial dysfunction. This dysfunction can be achieved either through the overexpression or repression of the expression levels of proteins associated with mitochondrial division (Wang et al., 2015; Xie et al., 2014).

Dynamin 1 (Drp1), also known as Drp1, is a key protein in maintaining the mitochondrial dynamic network (Jin et al., 2021). In the physiological state, it is localized in the cell cytosol and is regulated by a variety of protein translational modifications (PTMs) to adapt to different cellular environments, such as phosphorylation, ubiquitination, S-nitrosylation, palmitoylation, and SUMOylation (Jin et al., 2021). Drp1 is primarily composed of four distinct structural domains: the amino-terminal GTPase structural domain, the variable structural domain, the helical structural domain situated in the middle, and the carboxy-terminal GTPase effector structural domain (Mishra and Chan, 2016). The process of mitochondrial division encompasses the formation of both meso-regional and peripheral divisions. Mitochondrial division entails the formation of two spatially distinct types: midzone division, which occurs in the central region of the mitochondrion, and peripheral division, localized near its terminal regions (Kleele et al., 2021). Mitochondrial fission can be roughly divided into three steps, as follows: Drp1 protein is recruited from the cytoplasm and transferred to the outer mitochondrial membrane for oligomerization (Frohlich et al., 2013), which depends on its GTPase activity; hydrolysis of ATP to provide energy to allow Drp1 to form a ring structure; and the interplay between the endoplasmic reticulum and actin to drive the contraction of the Drp1 protein to reduce the molecular spacing, leading to the rupture of the OMM and IMM and ultimately triggering mitochondrial fission (Giacomello et al., 2020; Kalia et al., 2018). Upon completion of mitochondrial fission, the Drp1 helix detaches from the mitochondria and returns to the cytosol to undergo another mitochondrial division (Itoh et al., 2013).

One of the most characteristic post-translational modifications (PTMs) of the Drp1 is phosphorylation, which regulates mitochondrial fission. Depending on the specific phosphorylation site of the modification, Drp1 may exert either an activating or inhibitory effect on mitochondrial fission (Jin et al., 2021). Some of the phosphorylation sites that have been subjected to the greatest degree of study are Ser40, Ser44, Ser579, Ser585, Ser592, Ser616, Ser637, Ser656, Ser693, and other phosphorylation sites (Qi et al., 2019). It has been shown that mitochondrial fission is facilitated by the phosphorylation modifications of Ser40, Ser44, Ser579, Ser585, Ser592, and Ser616 as well as the dephosphorylation modifications of Ser637 and Ser656. Conversely, the phosphorylation modifications of Ser637 and Ser656 have been shown to inhibit mitochondrial fission. Phosphorylation at distinct Drp1 residues differentially regulates mitochondrial dynamics. While phosphorylation at Ser616 enhances fission activity, phosphorylation at Ser637 exerts an inhibitory effect under physiological conditions (Longo et al., 2021). Although Drp1-Ser637 phosphorylation predominantly suppresses mitochondrial fission under most physiological conditions, emerging evidence indicates its biological functions exhibit cell type-specific dependency and are dynamically regulated by microenvironmental signaling cues and upstream regulatory networks (Serasinghe and Chipuk, 2017). In diabetic patients and animal models, phosphorylation of the Drp1-Ser637 site in podocytes and endothelial cells in a high-glucose environment drives the translocation of Drp1 to mitochondria, which in turn promotes mitochondrial fission (Wang W. et al., 2012). In pathophysiological conditions, PKD-mediated phosphorylation of Ser637 has been demonstrated to enhance mitochondrial fragmentation (Jhun et al., 2018). A 2021 study revealed that phosphorylation at the Drp1 Ser637 site influences mitochondrial fission or fusion, contingent on the phosphorylation state of Ser616. It was demonstrated that phosphorylation at the Ser637 site stimulates Ser616 phosphorylation and that only the inhibition of downstream Ser616 phosphorylation results in mitochondrial elongation (Gonzalez et al., 2021). Concurrently, accumulating evidence demonstrates that Ser637 phosphorylation participates in circadian regulation of mitochondrial fission (Serasinghe and Chipuk, 2017), highlighting the need for systematic elucidation of its spatiotemporal regulatory mechanisms and precise molecular determinants governing mitochondrial fission dynamics.

FIS1, the earliest identified mitochondrial junction protein, resides on the outer mitochondrial membrane, where it facilitates mitochondrial fission by engaging in a complex with Drp1. In yeast, Fis1 interacts with the ligand protein Mdv1 to promote the synthesis and assembly of Drp1-GTP oligomers, which are involved in the regulation of mitochondrial asymmetric fission (Frieden et al., 2004). However, Mdv1 has not been detected in mammalian cells (Stojanovski et al., 2004), where FIS1 is required to interact with additional outer membrane receptors, including Mff, MID49, and MID51, to facilitate Drp1 polymerization and assembly into a helical or ring structure surrounding the mitochondrial outer membrane, thereby recruiting Drp1 to the division site, which ultimately initiates fission (Takamura et al., 2012). Mff can recruit Drp1 independently of Fis1 and is involved in the recruitment of Drp1 to the mitochondrial and peroxisomal membranes. MiD49 and MiD51 are chordate-specific mitochondrial elongation factor proteins, both of which can recruit Drp1 to the mitochondrial fission site independently of Fis1 and Mff and can also coordinate with Mff to regulate Drp1-mediated mitochondrial fission (Kraus and Ryan, 2017). Consequently, the relative levels of FIS1, Mff, MiD49, and MiD51 serve as crucial determinants of homeostatic mitochondrial dynamics (Yu et al., 2017).

As a mechanoenzyme, dynamin 2 (Dyn2) specifically engages in the terminal membrane scission phase of mitochondrial division following Drp1-mediated recruitment and oligomeric assembly at the OMM (Lee et al., 2016). Its functional execution strictly depends on GTPase activity to drive constrictive severing of the OMM (Mahecic et al., 2021). Concurrently, the mitochondrial signature lipid cardiolipin facilitates Drp1 oligomerization, synergistically enhancing coordinated constrictive remodeling of both OMM and IMM (Adebayo et al., 2021), thereby mechanistically advancing Drp1-dependent mitochondrial fission.

Recent breakthroughs have been made in the molecular mechanisms surrounding mitochondrial dynamics. Related studies have found that the endoplasmic reticulum is the initiating step in mitochondrial division, that the endoplasmic reticulum serves to mark the onset of mitochondrial contraction and division (Friedman et al., 2011), and that the lysosome’s location of contact with the mitochondrion is likely to be the site of initiation of division (Wong et al., 2018). Furthermore, prior to division, Drp1, Mff, and MiD49/51 bind specifically to endoplasmic reticulum-mitochondrial contact sites, where they facilitate mitochondrial membrane remodeling and division (Osellame et al., 2016; Yamashita et al., 2016).

**FIGURE 4:**
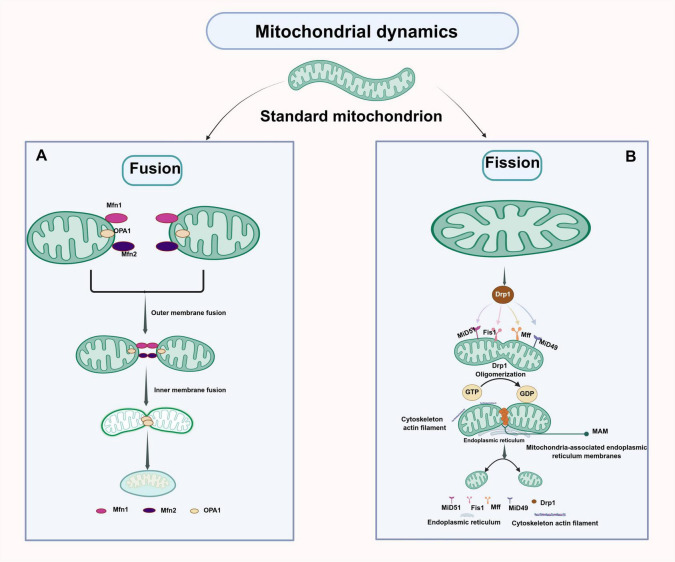
Schematic illustration of the mechanism of mitochondrial dynamics. **(A)** Mitochondrial fusion is governed by a set of membrane-anchored proteins, such as Mfn1, Mfn2, and Opa1. Mfn1 and Mfn2 facilitate the fusion of the outer mitochondrial membrane, while Opa1, the predominant mitochondrial DNA (mtDNA) in mammals, is responsible for the fusion of the inner mitochondrial membrane and the reshaping of mitochondrial cristae. **(B)** During mitochondrial division, Drp1 binds to its receptors (MFF, MID49, MID51, and FIS1) to form Drp1 oligomers, contracting the mitochondria to facilitate division. Figure was created with BioRender software.

In conclusion, MQC is an integrated network that monitors mitochondrial quality and works in concert to maintain mitochondrial homeostasis by coordinating various processes, including mitochondrial biogenesis, mitochondrial autophagic degradation, and the balance of mitochondrial dynamics (fission, fusion) (Zhou et al., 2023). The mitochondrial quality control regulatory mechanism is intricate and encompasses multilevel, multi-mechanism, and multipathway processes. Consequently, a comprehensive and systematic understanding of the specific molecular regulatory mechanisms of mitochondrial quality control using novel technologies and methodologies is a pivotal objective of future research (Figure 4).

## 4 Crosstalk between mitochondrial dynamics, biogenesis, and mitophagy

MQC is a dynamic and coordinated process in which mitochondrial biogenesis, mitophagy, and mitochondrial fission/fusion interact and regulate each other to maintain mitochondrial homeostasis. A growing body of evidence has demonstrated that the degradation of mitochondrial fission and fusion-related factors plays a pivotal role in regulating mitophagy (Catanzaro et al., 2019; Yang et al., 2022; Zhang et al., 2020). The equilibrium between mitochondrial dynamics and mitophagy is essential for the continuous renewal of mitochondria and the generation of new mitochondria (Gan et al., 2018). The maintenance of a balance between mitophagy and mitochondrial biogenesis is a prerequisite for cellular adaptation and resilience. However, an imbalance between the two results in cellular degradation and the initiation of cell death mechanisms (Palikaras and Tavernarakis, 2014). Consequently, the optimal functioning of mitochondria is maintained by orchestrating the intricate equilibrium between mitochondrial biogenesis, autophagy, and dynamics, including fission and fusion.

### 4.1 Co-regulation of mitochondrial biogenesis and mitophagy

Mitochondrial-related pathways are intertwined, convergent, and differentiated (Collier et al., 2023), and changes in mitochondrial number are closely related to mitochondrial biogenesis and mitophagy. Mitochondrial biogenesis renews or replaces damaged mitochondria through growth, while mitophagy is responsible for the removal of damaged or functionally defective mitochondria. The process of inter-regulation and inter-coordination of the two is an important pathway that influences the balance of mitochondrial numbers in the organism, or homeostasis. Mitophagy is responsible for the removal of damaged or defective mitochondria. Damage to mitochondrial biogenesis resulting from defective mitophagy may result in a decrease or increase in the number of mitochondria (Palikaras et al., 2015). Several studies have demonstrated that mitochondrial biogenesis is an important indicator for assessing mitochondrial function and mitophagy in disease states. Furthermore, these studies have shown that mitochondrial biogenesis is accompanied by mitophagy and that abnormal mitophagy inhibits mitochondrial biogenesis.

The PGC-1α-NRF1-FUNDC1 signaling pathway plays an important role in the balance between mitophagy and mitochondrial biogenesis (Liu L. et al., 2021). PGC-1α is a pivotal regulator of mitochondrial biogenesis and mitophagy (Palikaras and Tavernarakis, 2014). On the one hand, PGC-1α stimulates mitochondrial biogenesis by enhancing NRF1 transcriptional activity and TFAM gene expression. On the other hand, PGC-1α/NRF1 regulates FUNDC1-mediated mitophagy through transcription, promoting mitochondrial biogenesis during the process of mitochondrial autophagy, accelerating mitochondrial turnover, and maintaining normal mitochondrial respiration and mitochondrial population stability. Furthermore, mitochondrial biogenesis, which is regulated by PGC-1α, positively influences PINK1/Parkin-mediated mitophagy. In a cellular model of dopamine neurotoxicity induced by fisetinone, silencing of PGC-1α expression was found to significantly increase the protein expression levels of PINK1, parkin, and their phosphorylated proteins (Peng et al., 2019).

PINK1 and Parkin have been identified as regulators of multiple structural domains of mitochondrial quality control, with additional roles in mitochondrial biogenesis and mitophagy (Lee H. et al., 2017). The deletion or inactivation of Parkin down-regulates PGC-1α, which in turn leads to the selective degeneration of dopamine neurons in the substantia nigra pars compacta (SNpc). This process can be reversed by the overexpression of PGC-1α, which in turn restores mitochondrial biogenesis (Siddiqui et al., 2016; Stevens et al., 2015). The overexpression of Parkin in cortical neurons has been demonstrated to increase the levels of PGC-1α and mtDNA copy number (Zheng et al., 2017). A reduction in the expression of PINK1 was demonstrated to result in a significant decline in the function of the mitochondrial electron transport chain (ETC) as well as the mtDNA copy number in hepatocellular carcinoma cell lines, as evidenced in a study (Kung-Chun et al., 2019). PINK1 and parkin can positively regulate mitochondrial biogenesis through the PARIS/PGC-1α axis (Pirooznia et al., 2020). It has been observed that PARIS (ZNF746) is a transcriptional repressor of PGC-1α and NRF1, as well as a common substrate for PINK1 and parkin. Furthermore, PINK1/Parkin mediates PARIS degradation through ubiquitylation, which in turn is involved in the regulation of mitochondrial biogenesis (Lee H. et al., 2017). In neuronal cells, following the knockdown of PINK1 and Parkin, PINK1 is observed to promote PARIS ubiquitination and Parkin proteasomal degradation through phosphorylation of Parkin and PARIS. Consequently, this results in a reduction in the expression of PGC-1α (Pirooznia et al., 2020). However, some reports present an opposing view. Emerging evidence reveals reciprocal antagonistic regulation between PINK1/Parkin-mediated mitophagy and mitochondrial biogenesis in rotenone-induced *in vivo* and *in vitro* models. PINK1 silencing upregulated the mitochondrial biogenesis regulators PGC-1α and mtTFA protein expression, concomitant with a significant increase in mtDNA copy number. Conversely, PINK1 overexpression suppressed these biogenic markers (PGC-1α, mTFM protein levels, and mtDNA content) (Peng et al., 2019). These findings collectively indicate that PINK1 and Parkin play a role in the generation of new mitochondria by regulating PGC-1α levels.

The interaction between PINK1/Parkin and PGC-1α appears to co-regulate mitochondrial biogenesis and mitophagy, thereby maintaining mitochondrial homeostasis and mitochondrial quality control. However, the relationship between the mutual coordination of PINK1/Parkin and PGC-1α remains controversial. The experimental results presented above indicate that mitophagy and mitochondrial biogenesis are closely intertwined, with the interaction between these two processes playing a pivotal role in cellular adaptation and stress resistance (Palikaras and Tavernarakis, 2014). This section aims to provide a detailed account of the experimental study on the further investigation is required to elucidate the crosstalk between PINK1/parkin-mediated mitophagy and mitochondrial biogenesis, with the involvement of PGC-1α. Additionally, the regulatory mechanisms between other pathways or protein receptors mediating mitophagy and mitochondrial biogenesis require further investigation.

### 4.2 Co-regulation of mitochondrial biogenesis and mitochondrial dynamics

A crosstalk exists between mitochondrial biogenesis and mitochondrial fission/fusion. Studies have reported that PGC-1α not only regulates mitochondrial biogenesis, mitochondrial transcription and replication, and antioxidant systems but also participates in regulating mitochondrial fission and fusion and maintaining mitochondrial homeostasis (Zarch et al., 2009). The mitochondrial fusion/fission-associated proteins Mfn2 and Drp1 have been proposed to function as downstream nuclear transcription factors in the PGC-1α-mediated mitochondrial biogenesis process (Peng et al., 2017). Related studies have demonstrated that PGC-1α exerts a dual regulatory effect on mitochondrial dynamics. On the one hand, it positively regulates the mitochondrial fusion protein Mfn2, while on the other, it negatively regulates the expression of mitochondrial splitting-related proteins. Inhibition of PGC-1α expression has been shown to result in decreased expression of the mitochondrial fusion protein Mfn2, increased expression of p-Drp1, and increased mitochondrial fragmentation (Guo et al., 2015). An increase in PGC-1α expression was accompanied by a corresponding increase in the level of the fusion protein Mfn2, while the level of splitting protein levels was significantly reduced (Wang et al., 2019d). These results suggest that positive PGC-1α-Mfn2 regulation and negative PGC-1α-Drp1 regulation maintain the balance of mitochondrial dynamics. In a separate study, Ding et al. (2018) demonstrated that PGC-1α directly regulates Drp1 expression by binding to the upstream promoter, activates the SIRT1-PGC-1α signaling pathway, and markedly inhibits Drp1-mediated mitochondrial fission. Furthermore, it was demonstrated that PGC-1α has the capacity to repair damage to axonal mitochondrial transport and enhance mitochondrial swelling morphology. This may be attributed to the stimulation of the activity of the Mfn2 promoter (Soriano et al., 2006; Wang et al., 2022).

During the mitochondrial life cycle, mitochondrial fission and fusion can regulate both the equilibrium of mitochondrial dynamics and mitochondrial biogenesis. Peng et al. (2017) demonstrated that the application of a mitochondrial fusion promoter (M1) and an inhibitor of mitochondrial fission (Mdivi-1) resulted in a significant increase in the copy number of mtDNA. The findings suggest that mitochondrial fission and fusion may play a role in the regulation of mitochondrial biogenesis.

In conclusion, the above results collectively demonstrate that there is a crosstalk between mitochondrial division/fusion and transcriptional regulation of mitochondrial biogenesis, which is essential for maintaining mitochondrial homeostasis. In conclusion, the above results collectively demonstrate that there is a crosstalk between mitochondrial division/fusion and transcriptional regulation of mitochondrial biogenesis, which is essential for maintaining mitochondrial homeostasis.

### 4.3 The interaction between mitophagy and mitochondrial dynamics

A number of studies have demonstrated a correlation between mitochondrial dynamics and mitophagy (Zhou et al., 2015). In pathological conditions, an imbalance or abnormality of mitochondrial dynamics leads to morphological changes and dysfunction of mitochondria, which in turn triggers mitophagy. Mitophagy maintains the normal number and function of mitochondria by removing damaged mitochondria. The interaction between the two coordinates the balance of energy metabolism in cells (Wai and Langer, 2016; Yoo and Jung, 2018). Mitochondrial fission and fusion represent a pivotal step in the initiation of mitochondrial autophagy (Zhang et al., 2020). Mitochondrial fission results in the separation of damaged mitochondria, increases mitochondrial fragmentation, initiates the process of mitophagy, and provides the content for mitophagy (Otera and Mihara, 2011). Fusion between healthy and damaged mitochondria dilutes the damaged mitochondria into the healthy mitochondrial lattice, which in turn maintains overall mitochondrial health (Twig et al., 2008). The inhibition of mitochondrial fusion affects mitochondrial fission, which in turn enables the indirect regulation of mitochondrial autophagy.

A relationship exists between proteins that regulate mitochondrial fission and fusion and the PINK1/Parkin pathway, which is involved in the regulation of mitophagy. Additionally, Pink1 may be involved in the regulation of mitochondrial fission and fusion homeostasis independently of mitophagy. It has been demonstrated that PINK1 plays a regulatory role in the expression of key fission proteins, including Drp1, Fis1 and fusion protein Mfn2. These findings indicate that PINK1 affects the balance of mitochondrial fission/fusion processes (Peng et al., 2019). Furthermore, it has been shown that PINK1 mediates Drp1-Ser616 phosphorylation, promotes mitochondrial division and increases mitochondrial density, while simultaneously participating in the regulation of mitochondrial dynamics (Han et al., 2020). Drp1, a critical factor in mitochondrial fragmentation, plays a central role in the regulation of PINK1/Parkin-mediated mitophagy (Li et al., 2019). Inhibition of Drp1 expression or reduction of Drp1 activity significantly reduces the number of mature autophagosomes and inhibits the formation of autophagic microsomes, and mitochondrial fragmentation and Parkin translocation are significantly suppressed. Conversely, Drp1 overexpression was found to promote mitochondrial fragmentation and mitophagy. In addition, phosphorylation of Drp1 has been shown to play a role in the regulation of mitophagy (Chen et al., 2023; Ikeda et al., 2015). Pathological studies in degenerating neurons have demonstrated that S-nitrosylation of Drp1 specifically mediates aberrant mitochondrial recruitment, thereby triggering cascade activation of PINK1/PARKIN pathway-mediated mitophagy (Li et al., 2019). Furthermore, the autophagy receptor FUNDC1 has been shown to induce mitochondrial fragmentation, in addition to its involvement in the essential role of mitophagy (Dirks-Naylor et al., 2014). Research has indicated a correlation between mitochondrial fission protein Drp1 and the promotion of excessive mitophagy. This process involves Drp1 binding to the mitophagy receptor protein FUNDC1 (Yang et al., 2022), which subsequently results in ATP depletion and a significant decline in mitochondrial mass (Wang and Zhou, 2020). In addition to the observed increase in mitochondrial fragmentation, the overexpression of FIS1 has also been demonstrated to result in mitochondrial dysfunction and an increase in autophagosome formation (Gomes and Scorrano, 2008). In addition, mitochondrial fusion proteins play a pivotal role in the regulation of mitochondrial autophagy. Mfn2, a mitochondrial fusion protein, is phosphorylated by PINK1, which promotes the recruitment of Parkin to the outer mitochondrial membrane and initiates Parkin-dependent mitophagy (Chen and Dorn, 2013). It was observed that the inhibition of OPA1 expression and the subsequent reduction in mitochondrial fusion facilitated the isolation of dysfunctional mitochondria, which proved to be a more beneficial approach for the timely triggering of mitophagy and the removal of functionally impaired mitochondria (Twig et al., 2008). Research demonstrates that BNIP3/NIX-mediated mitophagy orchestrates mitochondrial dynamics homeostasis through dual regulatory mechanisms: by suppressing the activity of fusion protein Opa1 while enhancing fission protein Drp1 functionality to induce mitochondrial network fragmentation, thereby facilitating segregation of damaged organelles; concurrently, BNIP3 recruits Parkin to mitochondria to initiate autophagic flux, whereas NIX—as a ubiquitination substrate of Parkin—undergoes conformational remodeling post-modification to specifically engage adaptor protein NBR1, ultimately enabling targeted autophagic degradation of compromised mitochondria (Wang et al., 2023).

Taken together, mitochondrial biogenesis, fission/fusion, and mitophagy do not function independently; instead, they crosstalk to regulate mitochondrial network homeostasis and the quality control system. Furthermore, the crosstalk between the three in different organizations or cells, the manner in which this crosstalk occurs, and the specific molecular mechanisms involved require further in-depth study.

## 5 Abnormal mitochondrial quality control in neurodegenerative diseases

**FIGURE 5:**
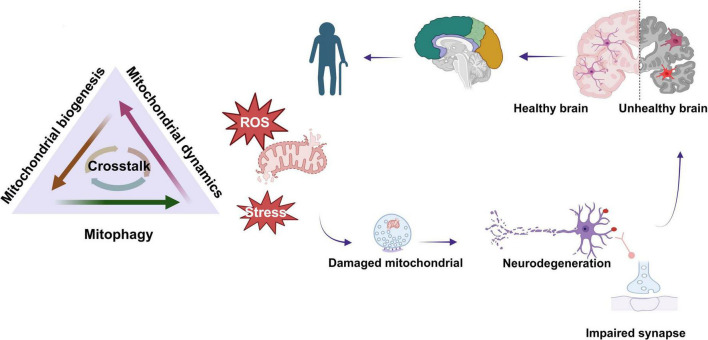
Schematic illustration of abnormal mitochondrial quality control in neurodegenerative diseases. Figure was created with BioRender software.

Neurodegenerative diseases are chronic neurological disorders that are characterized by selective neuronal degeneration and neuronal loss or death. The clinical symptoms of these diseases manifest as cognitive decline, memory loss, and impairment of limb movement. Emerging evidence strongly implicates mitochondrial dysfunction as a central pathogenic mechanism in neurodegenerative disorders (Nguyen et al., 2019; Park et al., 2018; Wang et al., 2019d). Mitochondrial functional homeostasis serves as the molecular cornerstone for sustaining neuronal physiological activities (Li J. et al., 2023). As high-energy-demand cells, cerebral neurons require continuous bioenergetic supply to power neurotransmitter biosynthesis and vesicular secretion, action potential propagation, and synaptic plasticity modulation. Operating as neuronal bioenergetic hubs, mitochondria sustain neuronal excitability and synaptic transmission through ATP generated via oxidative phosphorylation, thereby providing obligatory energy substrates for these electrophysiological processes (Carinci et al., 2021). The mitochondrial quality control system achieves precision maintenance of neuronal metabolic homeostasis through spatiotemporal orchestration of mitochondrial network distribution and stringent preservation of functional fidelity. The relationship between mitochondrial quality control and neurodegeneration is currently a subject of intense research interest in the field. Several clinical studies have demonstrated that abnormalities in mitochondrial biogenesis, autophagy, fission, and fusion are observed in a range of inherited neurodegenerative diseases (Ashleigh et al., 2023; Pritam et al., 2022; Whitley et al., 2019). However, under pathological conditions, mitochondrial dysfunction and protein aggregation (e.g., Aβ, α-synuclein) engage in a mutually reinforcing vicious cycle, distinct from a unidirectional causal relationship. This study specifically focuses on delineating how mitochondrial quality control system dysregulation or functional deficits drive neuronal degeneration, rather than interrogating bidirectional regulatory mechanisms. This section summarizes current research progress on mitochondrial biogenesis, mitophagy, and mitochondrial dynamics in common neurodegenerative diseases (AD, PD, HD, and ALS) (Figure 5).

### 5.1 Alzheimer’s disease

Alzheimer’s disease is a common neurodegenerative disease with clinical symptoms including cortical degeneration with severe memory loss, cognitive impairment, and behavioral abnormalities. The pathological features of Alzheimer’s disease include the abnormal deposition and accumulation of β-amyloid (Aβ) and phosphorylated tau (p-tau) in neurons, which induce neurogenic fiber tangles, leading to impaired synaptic and cholinergic neuronal function (Ashleigh et al., 2023; Pritam et al., 2022). The pathogenesis of Alzheimer’s disease is complex and not yet fully defined. It has been reported that mitochondria are one of the central players in the pathogenesis of AD and that mitochondrial dysfunction is a driver or predisposing factor in the onset or progression of AD (Sharma C. et al., 2021; Swerdlow, 2018). Furthermore, it has been identified as an early metabolic change and a prominent feature of the disease (Swerdlow, 2018). The aberrant accumulation of Aβ and hyperphosphorylated Tau proteins on mitochondria results in mitochondrial respiratory chain complex I activity, impaired mitochondrial membrane potential (MMP), and massive production of reactive oxygen species (ROS), which in turn affects mitochondrial function (Guha et al., 2020). It has also been proposed that impaired mitochondrial function acts in an inverse manner on ROS, resulting in inadequate bioenergy provision and oxidative stress. This, in turn, further exacerbates the accumulation of Aβ and tau, ultimately leading to impaired synaptic plasticity and cognitive impairment (Chakravorty et al., 2019). Consequently, impaired mitochondria play a pivotal role in the pathogenesis of AD. Further investigation into the mechanisms of mitochondrial dysfunction and impaired mitochondrial quality control may facilitate the identification of novel molecular targets for the development of new drugs for AD.

A number of studies have demonstrated that there are extensive mitochondrial abnormalities in the brains of AD patients and animal models (Swerdlow, 2018). Furthermore, significant changes have been observed in the content of various proteins involved in mitophagy, mitochondrial dynamics, and mitochondrial biogenesis (Quinn et al., 2020). Furthermore, the expression of genes associated with mitochondrial biogenesis, including PGC-1α, Nrf1, NRF2, and TFAM, was observed to have decreased in brain tissue samples from AD patients (Kerr et al., 2017). β site-APP cleaving enzyme 1 (BACE1) is a transmembrane aspartic protease and a rate-limiting enzyme for Aβ production. PGC-1α, as a key regulator of mitochondrial biogenesis and transcriptional regulator, is involved in the regulation of BACE1 transcription. It has been demonstrated that the production of PGC-1α protein is inversely proportional to the concentration of Aβ (Qin et al., 2009; Yang et al., 2023). The overexpression of PGC-1α adeno-associated virus in the brain region of APP23 model mice was observed to result in increased transcription of growth factors, reduced levels of BACE1, and diminished Aβ-mediated neuroinflammation, which collectively led to reduced β-amyloid production and neuronal loss (Katsouri et al., 2016). Furthermore, PGC-1α suppresses the transport of NF-κB p65 from the cytoplasm to the nucleus and the degradation of IκBα by regulating NF-κB, thereby reducing Aβ-induced neuronal death and inhibiting neuroinflammation, which in turn reduces mitochondrial damage and restores AD cognitive deficits (Zhang Y. et al., 2017). TFAM functions as a downstream effector of PGC-1α, regulating mitochondrial biogenesisx. TFAM binds to mtDNA and forms a nuclear-like structure, thereby protecting mtDNA from the adverse effects of Aβ toxicity and oxidative stress, inhibiting the vicious cycle of neuronal mitochondrial dysfunction, and thus improving the pathophysiology of Alzheimer’s disease (Oka et al., 2016).

Mitophagy, as an essential mitochondrial quality control mechanism, plays a pivotal role in maintaining neuronal health and function. Studies have demonstrated that defective mitophagy is responsible for the excessive accumulation of damaged mitochondria observed in the brain regions of AD patients. Furthermore, it was found that the basal level of mitochondrial phagocytosis was lower than 50% in the brain tissues of AD patients compared to the healthy population. Additionally, a 60% lower mitophagy ratio and a significant increase in the accumulation of damaged mitochondria were observed in the hippocampal brain region of APP/PS1 model mice (Fang et al., 2019). Moreover, the overexpression of the mitophagy proteins PINK1 and parkin in microglia was demonstrated to significantly inhibit STING-induced inflammation, reduce the levels of insoluble Aβ _1–42_ and Aβ _1–40_ content, and ameliorate the cognitive deficits in the AD mouse model (Fang et al., 2019). The restoration of mitophagy has been demonstrated to facilitate the reduction of Aβ plaques, the elimination of tau hyperphosphorylation, and the improvement of cognitive dysfunction. Xie et al. (2022) demonstrated that the activation of neuronal activity and the elimination of Aβ and tau proteopathies by oral administration of drugs promoting mitophagy were effective in improving the cognitive deficits in both the nematode and rodent models of AD. Impaired mitophagy may also be attributed to impaired autophagosome-lysosome fusion. It has been demonstrated that the amyloid precursor protein-derived C-terminal fragment (APP-CTF) may act as a trigger for AD pathology, with its overaccumulation resulting in impaired mitochondrial structure and defective mitophagy. This is evidenced by inconsistent recruitment of PINK1/Parkin to the mitochondria, overaccumulation of LC3-I and/or LC3-II, and insufficient fusion of mitochondria with lysosomes (Vaillant-Beuchot et al., 2021). Under pathological conditions, FUNDC1 persists in a phosphorylated state that specifically impedes its molecular interaction with the autophagosomal marker protein LC3-II, consequently suppressing mitophagy initiation and ultimately inducing neuronal dysfunction (Biswal et al., 2018).

Mitochondrial fragmentation has also been demonstrated in AD patients and model animals. Excessive mitochondrial fission has been shown to reduce ATP synthesis by interfering with oxidative phosphorylation complex assembly and disrupting the integrity of mitochondrial cristae, ultimately resulting in impaired neuronal function (Blagov et al., 2022). Wang et al. (2017) the researchers observed mitochondrial fragmentation and ultrastructural damage in the brains of APP transgenic mice using confocal microscopy and electron microscopy. Additionally, they found that mitochondrial dynamics abnormalities were present in the early stages of AD development. Biochemical assessments of peripheral blood from AD patients demonstrated significantly elevated levels of the mitochondrial fission factor Fis1, with postmortem brain specimens further revealing upregulated Drp1 expression in AD cases (Bera et al., 2022; Pakpian et al., 2020). Leveraging the GEO dataset GSE173955 (containing RNA-sequencing [RNA-seq] data derived from hippocampal tissues), Han et al. (2024) conducted bioinformatic analyses revealing marked downregulation of mitochondrial fusion regulators OPA1 and Mfn2 in AD groups compared to non-AD controls. Studies further elucidate pathological interactions between the mitochondrial fission regulator Drp1, phosphorylated tau (p-tau), and Aβ. Aβ accumulation induces excessive free radical generation, which activates Drp1 and its cofactor Fis1, resulting in impaired mitochondrial trafficking to synapses and significantly reduced synaptic ATP production. These alterations ultimately cause synaptic dysfunction in AD rat models. Concurrently, elevated p-tau levels induce reactive ROS overproduction and enhance GTPase activity of Drp1, directly driving pathological mitochondrial hyperfission and functional collapse in neurons (Bhatti et al., 2023; Pradeepkiran and Reddy, 2020).

Dysregulated mitochondrial dynamics compromise neuronal function via neuroinflammatory pathways (Zhang M. et al., 2021). In AD animal models, Drp1 orchestrates early inflammatory responses in oligodendrocytes and microglia. Aberrant activation of Drp1 disrupts mitochondrial homeostasis, thereby triggering NLRP3 inflammasome activation and caspase-3 cleavage, which in turn amplify neuroinflammatory cascades (Elsherbini et al., 2020; Sbai et al., 2023). These pathological processes exacerbate Aβ deposition and tau-mediated neurodegeneration, ultimately leading to neuronal death or functional deficits. Mechanistically, hyperactivation of Drp1 suppresses hexokinase 1 (HK1), a glycolytic enzyme localized to the mitochondria, inducing metabolic reprogramming that promotes NLRP3-driven inflammation and oligodendrocyte pyroptosis (Pradeepkiran and Reddy, 2020; Zhang M. et al., 2021). Concurrently, Drp1-mediated mitochondrial fragmentation significantly reduces synaptic ATP production, directly impairing synaptic transmission efficacy.

To conclude, both AD populations and animal models have been demonstrated to exhibit abnormalities in relevant proteins that regulate mitochondrial biogenesis, mitophagy, and mitochondrial dynamics. Furthermore, these interactions between these proteins and Aβ, p-tau, and other related factors ultimately influence neuronal function and lead to cognitive deficits, as observed in AD.

### 5.2 Parkinson’s disease

Parkinson’s disease is the second most common chronic and late-onset neurodegenerative disease after Alzheimer’s disease, with clinical features including slowness of facial expression, increased muscle tension throughout the body, bradykinesia, progressive resting tremor, and dementia (Heusinkveld et al., 2018). The pathological process can be defined as the depletion of dopamine-producing neurons located in the substantia nigra pars compacta (SNpc), a region within the midbrain (Bloem et al., 2021). Nevertheless, the intricacies of its pathogenesis remain to be fully elucidated, necessitating further research. It has been demonstrated that the abnormal accumulation of Lewy bodies containing α-synuclein (α-Syn) is a significant pathological hallmark of PD (Dorsey et al., 2018). Furthermore, patients with advanced PD are at risk of developing dementia as a result of the accumulation of α-Syn in Lewy bodies. A number of studies have demonstrated that α-Syn is a presynaptic protSein that is highly enriched in presynaptic nerve endings. It is located in mitochondria or other organelles in the majority of neurons and is involved in the regulation of synaptic vesicle transport and endocytosis in neurons. Furthermore, α-Syn plays a key role in the causative factors of familial and sporadic PD (Del and Braak, 2016; Nishioka and Hattori, 2020). A growing number of studies now indicate that mitochondrial dysfunction is an important factor in the pathogenesis of PD and a causative element at the core of familial and sporadic PD. It has been observed that mtDNA loss, mitochondrial damage, or defective mitochondrial function affect dopaminergic neurons in patients with PD (Zhang L. et al., 2021). In the meantime, studies have confirmed that there is a bidirectional regulatory interaction between α-Syn and mitochondrial dysfunction, which leads to structural alterations and functional defects in mitochondria (Li H. Y. et al., 2023).

PGC-1α, a pivotal regulator of mitochondrial biogenesis and cellular resistance to oxidative stress, plays a pivotal role in the function and survival of dopaminergic neurons in the substantia nigra (Halling and Pilegaard, 2020). In response to stress, low expression of PGC-1α results in a reduction in ATP synthesis and an increase in ROS production, which in turn leads to dopamine neuron loss or damage. The current body of research has demonstrated a potential correlation between mitochondrial production and PD. Furthermore, a deficiency of PGC-1α has been identified as a factor that inhibits the activity and function of dopaminergic neurons and causes behavioral dysfunction in the mouse central nervous system (Kuczynska et al., 2021). Zhou et al. (2019) demonstrated that activating PGC-1α-dependent signaling cascades enhances mitochondrial biogenesis, which confers dopaminergic neuroprotection through upregulation of tyrosine hydroxylase (TH) expression. Neuropathological investigations in the substantia nigra pars compacta of Parkinson’s disease patients demonstrate that deficient PGC-1α expression compromises mitochondrial biogenesis and disrupts the coordinated transcriptional activation of antioxidant stress-responsive gene networks, thereby precipitating dopaminergic neuronal degeneration (Piccinin et al., 2021). Intervention studies employing dopaminergic neuron-targeted PGC-1α overexpression via adeno-associated viral vectors reveal enhanced redox homeostasis in striatal neurons and marked attenuation of programmed dopaminergic cell death, establishing this transcriptional coactivator’s neuroprotective capacity in PD pathophysiology (Wang et al., 2019c). Furthermore, it has been reported that PGC-1α may also inhibit rotenone-induced dopaminergic neurotoxicity by regulating the dynamic balance of mitochondrial fission and fusion proteins and determining the structure of the mitochondrial network (Peng et al., 2017). Alternatively, PGC-1α may mediate the expression of motor proteins in spinal cord motoneurons and thus modulate movement disorders in an animal model of PD through the stimulation of the upstream promoter of the mitochondrial fusion-associated protein Mfn2 (Mou et al., 2021; Soriano et al., 2006). Consequently, PGC-1α exerts a regulatory influence on mitochondrial biogenesis, which in turn affects the function of PD dopamine neurons. It is possible that this process may modulate PD pathophysiological factors by participating in multiple pathways (Guo B. et al., 2024).

A number of studies have demonstrated a close association between PD-related genes and mitochondrial integrity. Mutations in genes such as PINK1 (PARK6) and PARKIN (PARK2) are the most common cause of a form of Parkinson’s disease that is difficult to diagnose and one of the earliest mutation-associated genes in familial autosomal recessive inheritance of PD (Cooper et al., 2017; Kitada et al., 1998). It has been demonstrated that both Parkin and PINK1 are involved in the regulation of mitophagy. Several studies have demonstrated that defective mitophagy is accompanied in the amygdala of patients with Parkinson’s disease (Cai and Jeong, 2020; Chan, 2020). PINK1 may interact reciprocally with α-Syn, and studies have reported that overexpression of PINK1 in cells removes excess α-Syn, which in turn prevents mitochondrial defects and apoptosis and reduces neurotoxicity induced by α-Syn (Liu Z. Q. et al., 2021). The accumulation of α-Syn in excess also activates the autophagy-lysosomal pathway (Gu et al., 2024; Zhang et al., 2018), mutations in α-Syn proteins undergo misfolding in transcription-translation to produce toxicity, and defective mitophagy further exacerbates neurotoxicity, leading to neuronal loss or damage (Ciron et al., 2015). Mutations in the Parkin gene have been shown to inhibit the ubiquitination of synaptic binding protein 11 (Synaptotagmin-11, Syt11), which in turn inhibits the endocytosis of dopamine neurons. This ultimately leads to a progressive loss of dopamine neuron function and neurotoxicity (Wang et al., 2018).

In addition to PARK6 (encoding PINK1) and PARK2 (encoding Parkin), other genes associated with Parkinson’s disease, such as PARK7 (encoding the DJ-1 protein) and PARK8 (encoding the LRRK2 protein), are also thought to be mutated in patients with Parkinson’s disease (Walter et al., 2019). Both the DJ-1 and LRRK2 proteins are associated with the autophagy-lysosome pathway, maintaining a role in mitochondrial and lysosomal function while being essential for maintaining normal cellular function and survival (Lizama and Chu, 2021). Genetic mutation or deficiency of PARK7/DJ-1 disrupts dopamine metabolism, induces ROS accumulation, and impairs mitochondrial function in neuronal cells. Mechanistically, this results from compromised regulation of mitochondrial homeostasis by DJ-1. Restoration of DJ-1 expression ameliorates these defects by enhancing mitochondrial electron transport chain activity and reducing ROS overproduction, thereby attenuating oxidative stress-mediated neuronal damage (Bonifati et al., 2003; McCoy and Cookson, 2011). Furthermore, the DJ-1-encoded proteins act downstream of the PINK1/Parkin pathway or possibly in a parallel pathway to the PINK1/Parkin pathway to maintain mitochondrial function (Kinnart et al., 2024; Thomas et al., 2011; van der Merwe et al., 2015). LRRK2 is a protein kinase that is predominantly located in the mitochondrial outer membrane (Biskup et al., 2006; West et al., 2005). The GTP-binding protein RAB10 (RAB10) serves as a substrate for LRRK2, which may represent a pivotal link between PINK1/Parkin and LRRK2 (Wauters et al., 2020). It was observed that the G2019S mutation in LRRK2 results in delayed mitochondrial arrest and increased phosphorylation of the RAB10 protein (at the threonine 73 site), which in turn leads to impaired PINK1/Parkin-mediated mitophagy (Wauters et al., 2020). Wang D. X. et al. (2021) demonstrated that activation of BNIP3-mediated mitophagy attenuates dopaminergic neuronal damage in both MPP^+^-tinduced cells and MPTP-induced Parkinson’s disease mouse models.

Mitochondria are highly dynamic organelles that undergo fusion and fission processes. Imbalance in mitochondrial fission and fusion is a key trigger for Parkinson’s disease, and defects in mitochondrial dynamics limit mitochondrial movement, leading to a reduction in ATP, excessive oxidative stress, and mtDNA deficiency, which ultimately leads to cell death (Geng et al., 2019). The role of Drp1 has been identified as a contributing factor in the pathogenesis of Parkinson’s disease (Wu et al., 2017), with a substantial body of evidence demonstrating the involvement of α-Syn, DJ-1, LRRK2, PINK1, and Parkin in the regulation of mitochondrial dynamics and homeostasis through the Drp1 pathway (Irrcher et al., 2010; Kamp et al., 2010). The accumulation of α-Syn in excess is known to interact with the mitochondrial outer membrane, resulting in mitochondrial fragmentation (O’Donnell et al., 2014; Pozo et al., 2017). On the one hand, α-Syn is involved in mediating mitochondrial fragmentation independently of Drp1. This is evidenced by the fact that endogenous α-Syn levels lead to mitochondrial fragmentation (Kamp et al., 2010), and α-Syn is forcibly delivered to the mitochondrial membrane, leading to mitochondrial fragmentation (Pozo et al., 2017). On the other hand, α-Syn and Drp1 play interdependent and co-ordinated roles in the process of mitochondrial fission. Krzystek et al. (2021) identified the N-terminus of α-Syn as a potential mediator of mitochondrial fragmentation via the mitochondrial fission factor Drp1. It is noteworthy that α-Syn is also localized in mitochondria-associated endoplasmic reticulum membranes (MAMs), which are contact sites between two organelles, the mitochondria and the endoplasmic reticulum (Guardia-Laguarta et al., 2014). Furthermore, they are involved in the regulation of calcium homeostasis, mitochondrial morphology and function, and autophagy, among other pathophysiological processes. Mutations in α-Syn cause imbalances in mitochondrial dynamics, particularly Drp1-mediated mitochondrial fission (Friedman et al., 2011).

It was observed that DJ-1 exerts a regulatory effect on Drp1, thereby facilitating mitochondrial division and protecting neurons from oxidative stress-induced damage (Qin et al., 2017; Wang X. et al., 2012). LRRK2 is a large multi-structural domain protein kinase present in the cytoplasm and associated with mitochondrial membranes (Mata et al., 2006). Studies have suggested that the leucine-rich repeat kinase 2 (LRRK2) protein may be specifically involved in regulating Drp1-mediated mitochondrial fission. Su and Qi (2013) demonstrated that the LRRK2 G2019S mutant in PD induces excessive mitochondrial fragmentation. Inhibition of Drp1 with the aid of P110 was found to reduce LRRK2 G2019S-induced mitochondrial fragmentation, excessive autophagy, and neuronal toxicity (Niu et al., 2012; Su and Qi, 2013).

In conclusion, the pathological features and pathogenic factors of PD include the excessive accumulation of α-Syn in the substantia nigra pars compacta or its gene mutation. Furthermore, the PGC-1α, PINK1, Parkin, and Drp1 proteins interact with α-Syn and other mutant genes to affect the morphology and function of dopamine neurons in the PD brain. Consequently, the neuroprotective effects of PGC-1α, PINK1, Parkin, and Drp1 on the substantia nigra pars compacta may represent promising targets for the development of drugs to treat PD. The prevention of α-Syn localization to mitochondria and the regulation of mitochondrial quality control represent potential strategies for the prevention of PD neurodegeneration (Di Maio et al., 2016; Pickrell and Youle, 2015).

### 5.3 Huntington’s disease

Huntington’s disease is a rare autosomal dominant hereditary neurodegenerative disease. Given that HD affects different regions of the brain, its typical clinical manifestations include motor and cognitive disorders, as well as mental and behavioral abnormalities. Additionally, motor abnormalities resulting from striatum dysfunction, which is characterized by progressive, dance-like movements, are also observed (Hu et al., 2021). The disease is caused by the amplification of the CAG trinucleotide repeat in the first exon of the Huntingtin gene on chromosome 4 (Stoker et al., 2022). The mutated protein mHtt is progressively accumulated in cells, with a particularly high concentration in the brain. This accumulation results in mitochondrial dysfunction, impaired synaptic structure and function, and an imbalance of protein homeostasis. This, in turn, leads to neuronal loss and damage to nerve cell function (Guedes-Dias et al., 2016). A number of studies have indicated that mHtt-induced mitochondrial dysfunction plays a significant role in the pathogenesis of HD (Yan et al., 2018). The neurotoxic effects of mHtt are mediated by the induction of mitochondrial defects, which in turn result in aberrant energy metabolism and HD-related neuronal dysfunction (Costa and Scorrano, 2012).

Studies have demonstrated that mHTT interacts with the PGC-1α promoter, directly impairs the activity of PGC-1α, and impedes the capacity of PGC-1α to open downstream target genes (Cui et al., 2006; Hu et al., 2025). This results in a decrease in mitochondrial transcription factor TFAM and impaired mitochondrial function, which ultimately leads to increased vulnerability to oxidative stress and neuronal deterioration (Sharma A. et al., 2021). Thau et al. (2012) demonstrated that the accompanying progressive muscle atrophy and morphological abnormalities of the neuromuscular junction in HD mice may be associated with impaired expression of PGC-1 and its target genes. This was evidenced by *in vivo* studies, which revealed a markedly reduced expression of PGC-1α and its target genes in the striatum and muscle of HD mice (Chaturvedi et al., 2009). The aforementioned results indicate that PGC-1α, a co-regulator of mitochondrial biogenesis, energy homeostasis, and antioxidant defenses, may be a potential target for therapeutic intervention in HD (Di Cristo et al., 2019).

A number of *in vivo* studies have demonstrated that mHtt is involved in the process of mitophagy or regulates intermediately important aspects in animal models of HD. For example, it has been demonstrated that mHtt inhibits the delivery of the mitophagy receptor to autophagosomes, while simultaneously blocking autophagosome formation, disrupting mitophagy initiation, and affecting autophagosome translocation to lysosomes (Franco-Iborra et al., 2021). This results in defects in mitophagy, whereas a large number of dysfunctional mitochondria are not removed in time and excessively accumulate in neuronal cells, which induces a vicious circle-chain reaction (Wong and Holzbaur, 2014). It was observed that the overexpression of PINK1 in a Drosophila model of HD resulted in the amelioration of Parkin-mediated mitophagy defects and the attenuation of mHtt-induced neurotoxicity. Furthermore, it was demonstrated that the overexpression of PINK1 promoted neuronal activity and protected neuronal integrity (Khalil et al., 2015). Consequently, mitophagy serves to protect neurons from damage in HD, and a deficiency in this process contributes to further malignant deterioration of HD pathology.

A substantial body of evidence indicates the presence of mitochondrial fragmentation in neuronal cells in the brains of HD patients, in which the expression levels of the mitochondrial fission/fusion proteins Drp1, Fis1, and Mfn are significantly altered (Davies et al., 1997; Shirendeb et al., 2012). The presence of mHtt in neuronal cells in the brains of HD patients has been shown to induce mitochondrial hyperfragmentation and to affect the mitochondrial respiratory chain complex by disrupting the balance of mitochondrial dynamics, which in turn induces impairment of mitochondrial function (Cherubini et al., 2020). Sawant et al. (2021) demonstrated that mHtt binds to the proteins Mfn and Drp1, which are involved in mitochondrial fusion and fission. This binding enhances the activity of GTPase Drp1, resulting in mitochondrial fusion and fission imbalances, mitochondrial distribution abnormalities, and mitochondrial axonal transport defects. These defects ultimately lead to impaired synaptic plasticity (Sawant et al., 2021). Inhibition of Drp1 has been demonstrated to restore mitochondrial and neuronal dysfunction in mHtt-induced HD models.

Taken together, mHtt can impede the dynamic alterations of mitophagy, mitochondrial biogenesis, and mitochondrial fission/fusion, resulting in the disruption of mitochondrial structure and function. This process may be a significant contributor to the pathogenesis of HD.

### 5.4 Amyotrophic lateral sclerosis

Amyotrophic lateral sclerosis is a progressive and fatal neurodegenerative disease that is also known as motor neuron disease. From a pathological perspective, the disease is characterized by the progressive degeneration of both upper motor neurons (UMN) in the cerebral cortex and lower motor neurons (LMN) in the brain stem and spinal cord. The clinical symptoms that result from this process include progressive muscle weakness, atrophy, speech and swallowing difficulties, as well as respiratory complications (Mejzini et al., 2019; van Eijk et al., 2022). Amyotrophic lateral sclerosis is a progressive and fatal neurodegenerative disease that has been classified by the World Health Organization (WHO) as one of the top five terminal diseases. The pathogenesis of ALS is approximately 90% uncertain. Studies have reported that more than 210 gene mutations contribute to the progression of ALS, with mutations in superoxide dismutase 1 (SOD1), fusion sarcoma gene (FUS), TAR DNA binding protein 43 (TDP-43), and other genes being recognized as risk factors for the development of ALS (Alami et al., 2014). It is becoming increasingly evident that mitochondria may play a significant role in the pathophysiology of amyotrophic lateral sclerosis (ALS). Mitochondrial dysfunction has been proposed as a major determinant of the onset or progression of ALS and the prevalence of familial and sporadic cases of ALS. In addition, mutations in ALS-specific genes may further exacerbate ALS progression by impairing mitochondrial function through a variety of pathways (Smith et al., 2019).

It has been suggested that PGC-1α may be a male-specific disease-modifying factor in amyotrophic lateral sclerosis (Eschbach et al., 2013). In the ALS rodent model, the mitochondrial biogenesis regulator PGC-1α has been demonstrated to protect upper motor neurons, participate in the production of neuromuscular junctions in lower motor neurons, and regulate oxidative stress in sensory neurons (Kuczynska et al., 2021). The study revealed that the expression levels of PGC-1α and PGC-1α regulators were diminished in the brain and spinal cord of ALS patients and the spinal cord of ALS model mice. PGC-1α and its downstream regulatory factors (NRF-1, NRF-2, and TFAM) play a pivotal role in the regulation of the spinal cord, muscle, and adipose tissue of SOD1-G93A ALS model mice. Studies demonstrate that PGC-1α overexpression activates the NRF2/HO-1 signaling pathway, thereby enhancing mitochondrial antioxidant capacity to prevent spinal motor neuron loss. This mechanism concurrently suppresses skeletal muscle atrophy with secondary fibrosis and facilitates mutant SOD1 aggregate clearance, ultimately delaying disease progression and extending survival in ALS animal models (Wen et al., 2021).

Abnormalities in mitochondrial structure and defects in mitophagy have been identified in the neurons of patients with sporadic ALS (Rodriguez et al., 2012; Sasaki and Iwata, 2007). It has been demonstrated that mutant SOD1 accumulates in the mitochondrial membrane gap, thereby reducing the activity of the electron transport chain (ETC) complex. Consequently, this results in mitochondrial damage and the accumulation of damaged mitochondria at the axon terminals of neurons. This, in turn, results in a reduction in neuronal activity. It was demonstrated that in the spinal cord of the SOD1-G93A ALS mouse model, the mitochondrial receptor SQSTM1/p62 is recruited to the mitochondrial surface, where it activates mitophagy, whereas knockdown of the Parkin gene reduces the loss of motor neurons, thereby delaying the progression of disease in SOD1-G93A mice (Palomo et al., 2018). A reduction in mitochondrial activity was observed in a mouse model of the TDP-43 mutation, and PINK1 and Parkin-mediated mitophagy were found to be impaired following the overexpression of TDP-43 (Sun et al., 2018). Conversely, the overexpression of Parkin resulted in a reduction in the number of neurons lost in the motor cortex of TDP-43 model animals while also inhibiting the degenerative phenotype observed in hTDP-43 model animals (Hebron et al., 2014). Recent studies have demonstrated that FUNDC1 exerts critical neuroprotective effects in ALS mouse models. Activation of FUNDC1-mediated mitophagy facilitates the clearance of damaged mitochondria and enhances mitochondrial function, thereby reducing motor neuron apoptosis and ameliorating motor performance in experimental subjects (Guo X. et al., 2024).

It has been demonstrated that excessive mitochondrial fission or fragmentation is a factor in the pathology of amyotrophic lateral sclerosis (ALS). Furthermore, mitochondrial dynamics play an important role in the onset and progression of ALS (Liu et al., 2013). A number of studies have indicated that there may be an imbalanced state of mitochondrial dynamics in animals that have been genetically modified to develop ALS. This is thought to be due to the presence of SOD1 in the mitochondrial membrane gap (Ehinger et al., 2015; Sharma et al., 2016). SOD1-mutant mice exhibited mitochondrial ultrastructural pathologies in spinal cord and skeletal muscle tissues, manifesting as reduced mitochondrial length, cristae disorganization, and increased spherical fragmented mitochondria (Mendez-Lopez et al., 2021). The results of the study by Joshi et al. (2018) indicate that the administration of the selective peptide inhibitor P110 to SOD1-G93A model mice resulted in the inhibition of Drp1/Fis1 expression, which in turn led to a reduction in mitochondrial structural defects in motor neurons, an improvement in motor function and a reduction in muscle atrophy, and an increase in the survival rate of the model mice. Furthermore, models of SOD1 and TDP-43 mutation exhibited inhibitory properties against neurotoxicity through dephosphorylation of Drp1. It was demonstrated that the inhibition of Drp1 activity resulted in a reduction of cell death, as observed in studies where mutant SOD1 or TDP-43 were introduced into the system (Choi et al., 2020). Nemtsova et al. (2023) demonstrated that moderate Drp1 upregulation in ALS Drosophila models restores physiological mitochondrial distribution within axons, enabling efficient mitochondrial trafficking to nerve terminals and thereby meeting axonal energy demands. Notably, Drp1 expression modulation enhances mitophagic clearance of dysfunctional mitochondria, which improves mitochondrial bioenergetic capacity and attenuates motor neuron degeneration. These findings delineate Drp1’s dual regulatory role in mitochondrial dynamics and quality control, providing novel therapeutic avenues for ALS targeting mitochondrial proteostasis.

As with other neurodegenerative diseases such as Alzheimer’s disease, Parkinson’s disease, and Huntington’s disease, a significant number of fundamental studies have demonstrated that energy metabolism disorders resulting from mitochondrial dysfunction represent the primary pathophysiological phenotypes of amyotrophic lateral sclerosis (Sassani et al., 2020; Vandoorne et al., 2018). Mutations in specific genes associated with ALS interact with key regulators of mitochondrial biogenesis, mitophagy, and mitochondrial dynamics in a direct or indirect manner, affecting the activity or number of motor neurons and the susceptibility of neuromuscular junctions.

Taken together, the progressive elucidation and investigation of ALS-related disease-causing genes, the successful construction of disease models through the utilization of technologies such as exon sequencing and gene editing, and the further investigation of the potential molecular regulatory mechanisms between ALS mutant genes and mitochondria will facilitate the opening of a new avenue for the study of targeted therapy for ALS disease.

## 6 Conclusion and perspective

In recent years, the role of mitochondria as a specific therapeutic target for neurodegenerative diseases has attracted significant scholarly interest, emerging as a prominent research direction and marking substantial advancements. While significant breakthroughs have been achieved in multimodal modulation of mitochondrial biogenesis, mitophagy, and dynamics, persisting challenges include incomplete elucidation of molecular regulatory networks and genetic foundations, limitations of single-pathway intervention strategies in achieving multi-target coordination, and technical barriers imposed by the selective permeability of the blood-brain barrier (BBB) that hinder clinical translation of mitochondrial-targeted therapies (Meng et al., 2024).

By summarizing the molecular mechanisms of the regulatory pathways of the mitochondrial quality control system, it has been found that there are a large number of pathways mediating mitophagy. However, issues such as the relationship between the pathways, the existence of reciprocal regulatory roles of different pathways, and the rules of mitophagy receptor regulation under different physiological conditions remain to be resolved. It has also been observed that there are numerous studies on the mechanism of PINK1/Parkin-mediated mitophagy *in vitro*. However, there is still a lack of evidence that PINK1/Parkin mediates mitophagy *in vivo*. Furthermore, the relevance of this to the pathogenesis of disease is controversial (Li J. et al., 2023).

In the meantime, the majority of research on mitophagy in the nervous system has concentrated on the molecular pathogenesis and potential therapeutic targets, while there is considerable debate surrounding the targets of mitophagy, the exact mechanism of receptor-mediated mitophagy, and how to address it. With regard to mitochondrial dynamics, Ser637 is an inhibitory phosphorylation site for the mitochondrial dynamics-associated protein Drp1, which exhibits opposing effects in different cells. For example, Galvan et al. (2017) demonstrated that in podocytes, phosphorylation of Drp1 at the Ser637/656 (human/rat) site promotes mitochondrial fission. The mitochondrial fusion protein OPA1 exists in different forms, each of which plays a distinct role in mitochondrial dynamics. L-OPA1 is associated with mitochondrial fusion, whereas S-OPA1 is linked to mitochondrial fission. This suggests that there is still scope for further investigation into the roles of Drp1 and OPA1 in disease (Ayanga et al., 2016). The relationship between the mutual regulation of mitophagy, mitochondrial dynamics, and mitochondrial biogenesis is also controversial. It is yet to be determined whether the balance of mitochondrial biogenesis regulated by PGC-1α and mitochondrial dynamics involved in Drp1 positively regulates PINK1/PARKIN-mediated mitophagy. Furthermore, in studies of ALS, mitophagy has been demonstrated to protect neurons from damage during the initial stages of disease progression. However, if it persists for a long period of time, causing excessive mitophagy, it can exacerbate neuronal damage (Jetto et al., 2022). The pathogenesis of PD and AD may be a feedback loop in which apoptosis of neuronal cells leads to mitochondrial dysfunction, which in turn exacerbates apoptosis. It is not yet clear whether impaired mitochondrial structure or function is the causative factor or the outcome. Therefore, further research is needed to address these questions (Killackey et al., 2020).

In view of the significant heterogeneity observed in mitochondria, it is essential to employ comprehensive methodologies, encompassing both domestic and international gene technology, multi-omics technology, single-cell sequencing, and other techniques. In the future, the overall regulatory network of mitochondrial homeostasis in this disease will be elucidated on a macroscopic scale, while the physiological and pathological regulatory mechanisms of mitochondria under various factors will be further elucidated on a microscopic scale. It is also necessary to clarify the interconnectivity between disparate pathways within the internal milieu of the mitochondrial quality control system, to analyze the role of mitochondria and neurons in modulating the functionality of the brain and central nervous system, and to propose novel concepts and methodologies for the early diagnosis, prevention, and treatment of neurodegenerative diseases. Moreover, the identification of novel therapeutic targets is crucial for the development of innovative and effective treatment modalities.

Furthermore, although the topic of this review is mitochondria, dysregulation of other organelles such as autophagosome, lysosomes and UPS commonly make contributions for the pathological progress of neurodegenerative diseases. For example, mitophagy mediated MQC heavily depends on efficient lysosome degradation, whose dysregulation reversely suppresses mitochondrial function, so they all interplay by each other to form a vicious cycle, participating in the pathogenesis of neurodegenerative diseases. Therefore, any therapeutic strategies to break the vicious is supposed to benefit for slowing down pathological progress, even curing diseases. Exploring the interplay between mitochondria and other organelles is a critical area for future research, which could inform therapeutic strategies and interventions for neurodegenerative diseases.
